# Examining Practitioner Competencies, Organizational Support and Barriers to Engaging Fathers in Parenting Interventions

**DOI:** 10.1007/s10578-017-0733-0

**Published:** 2017-05-18

**Authors:** L. A. Tully, D. A. J. Collins, P. J. Piotrowska, K. S. Mairet, D. J. Hawes, C. Moul, R. K. Lenroot, P. J. Frick, V. A. Anderson, E. R. Kimonis, M. R. Dadds

**Affiliations:** 1 School of Psychology, University of Sydney, Sydney, Australia; 2 School of Psychiatry, Faculty of Medicine, University of New South Wales, Sydney, Australia; 3 Learning Sciences Institute of Australia, Australian Catholic University, Brisbane, Australia, & Department of Psychology, Louisiana State University, Baton Rouge, USA; 4 Royal Children’s Hospital, Murdoch Children’s Research Institute, Departments of Psychology & Paediatrics, University of Melbourne, Melbourne, Australia; 5 School of Psychology, University of New South Wales, Sydney, Australia

**Keywords:** Parenting interventions, Father engagement, Practitioner competence

## Abstract

Evidence-based parenting interventions have been developed and evaluated largely with mothers. This study examined practitioner reports of rates of father attendance, barriers to engagement, organizational support for father-inclusive practice, participation in training in father engagement, and competencies in working with fathers. It also explored predictors of practitioner competence and rates of father attendance. Practitioners (*N* = 210) who delivered parenting interventions completed an online survey. Participants reported high levels of confidence in engaging fathers, but only one in three had participated in training and levels of father attendance in parenting interventions were low. Logistic regressions showed that high levels of practitioner competence were predicted by participation in training. Moderate levels of father attendance (vs. low levels) were predicted by greater number of years of experience while high levels of attendance (vs. low levels) were predicted by greater experience, higher levels of competence and higher levels of organizational support. The implications of the findings to informing policy and practice for enhancing father engagement are discussed.

## Introduction

Over the past decade, several reviews have concluded that fathers are underrepresented in both research and practice, across child welfare services [1–3] and mental health interventions for children and their families [4–8]. Evidence-based parenting interventions, which focus on enhancing the quality of parenting, are the most effective early interventions for reducing child externalizing behaviors (e.g., temper tantrums, aggression and oppositional behavior) and improving well-being [9, 10]. However, the effectiveness of these interventions is tempered by the low levels of father engagement, as research indicates enhanced child and parenting outcomes when fathers participate [11]. Practitioners who deliver parenting interventions are an important source of information regarding fathers and represent a critical target for intervention in efforts to increase father engagement. This paper describes the findings of a survey of practitioners to examine their competencies in engaging fathers in parenting interventions, as well as organizational practices and reported barriers to engagement. It also explores predictors of practitioner competencies and rates of father engagement.

The majority of studies on parenting interventions do not report rates of father participation [4, 6, 8]. Where rates are reported, only around 13–20% of attendees are fathers [12, 13]. Importantly, however, there is evidence that including fathers in interventions leads to improved outcomes for children. In a meta-analytic review (*k* = 26), Lundahl et al. [11] found that father engagement in parenting interventions was associated with improved parenting and reductions in children’s externalizing behavior in the short-term, but not long-term. However, other research has found long-term improvements in outcomes for children when fathers are included [14, 15]. It is not surprising that father engagement improves the effectiveness of parenting interventions, as inclusion of fathers (and the core parenting team) is likely to be necessary for: (1) addressing father-specific (as well as mother-specific) risk factors (e.g., harsh, coercive parenting) that may cause or maintain child externalizing problems; (2) reducing parenting conflict, which is in itself a key risk factor for child externalizing problems [16]; and (3) enhancing inter-parental consistency in implementation of parenting strategies.

The marginalization of fathers in parenting interventions is of concern for reasons other than intervention effectiveness. Sidelining fathers in parenting intervention research and practice suggests deeply ingrained assumptions about the lesser importance of fathers relative to mothers, known as the ‘deficit model’ of fathering [17, 18]. However, given the notable research confirming that fathers can have significant positive (and negative) influences on child social, educational and behavioral outcomes [19], a paradigm shift in father engagement is urgently needed. Although ‘engagement’ has been defined in various ways, a recent conceptual model called CAPE [20] defines engagement across several stages from Connecting (enrolment in a program), Attending (presence at sessions), and Participating (active participation), to Enacting (implementing the parenting strategies).

There is a paucity of research examining the reasons for the low rates of father engagement in services and interventions, especially in relation to parenting interventions across each of these stages. There are likely to be a range of interrelated factors that may act as barriers to father engagement. These include *practical factors* such as fathers’ work commitments and availability of child care; *personal factors* such as fathers’ knowledge about the program and beliefs about help-seeking and parenting; and *family factors* such as the extent to which mothers facilitate father engagement [21, 22]. *Practitioner factors* and *organizational factors* are also likely to be critical to rates of father engagement in parenting interventions. Practitioner factors include competencies such as knowledge, skills and attitudes [23] in engaging fathers, and organizational factors include offering sessions outside working hours, and policies and practices regarding father inclusion [22].

Practitioners who deliver parenting interventions are a valuable source of information about father engagement. Not only can they report on their own competencies in engaging fathers as well as their organization’s support for father-inclusive practice, but they can also provide valuable information about current rates of father engagement and perceived barriers to father engagement. Clearly, fathers are likely to be the best reporters of barriers to engagement, and we have recently conducted a survey of fathers to examine barriers to their participation in parenting programs [24]. However, it is important to contrast practitioners’ perspectives with those of fathers, to help elucidate any differing perceptions of barriers to engagement. For example, differences in perceptions between fathers and practitioners may indicate areas of misinformation or misunderstanding about fathers that could be targeted in future training programs. Practitioners are also likely to have specific knowledge in relation to certain barriers to father engagement, such as their own organizational barriers.

There have only been a few surveys of practitioners on the topic of father engagement conducted to date. These have examined rates of father engagement and factors associated with father engagement [25–27]; practitioner competencies and factors associated with these competencies [22, 25, 26, 28]; characteristics of interventions delivered to fathers [27]; and perceptions about effective strategies for father engagement [22, 27]. Overall, practitioners have reported low rates of father engagement in services and interventions. For example, Lazar et al. [26] found practitioners (psychologists and social workers) reported that fathers were involved in only one out of every five treatment sessions, and had significantly lower levels of involvement than mothers. Similarly, Scourfield et al. [27] found practitioners working in social services reported that only 21% of attendees at services were male. Duhig et al. [25] found family therapists reported rates of father engagement ranging from 21.2 to 39.5%, with fathers significantly less likely than mothers to attend when invited.

These studies have also examined both practitioner factors and organizational factors as predictors of father engagement and practitioner competencies in engaging fathers. Duhig et al. [25] found practitioners’ years of clinical practice and some aspects of continuing education (such as completion of seminars and reading books and journal articles in the past year) were associated with greater involvement of fathers in family therapy. Similarly, Fletcher et al. [28] found more years of practice and higher level of education were associated with higher levels of practitioner competence in a sample of family dispute resolution workers. Lazar et al. [26] found a higher number of family-related courses completed in the past year was associated with increased involvement of fathers in the family context (that is, with mothers and children), but not with fathers alone. However, this study also found that greater years of experience was associated with lower levels of father engagement (both fathers alone and in the family context). Female practitioner gender was associated with a lower number of father-only sessions, but not a lower rate of father engagement in the family context [26]. Other research has also failed to find an association between practitioner gender and rates of father engagement [25] or practitioner competence [28]. Organizational factors such as service-level commitment to involving the entire family and flexible working hours were both associated with greater engagement of fathers in the family context (but not fathers alone) [26], but again, not all research has found this association [25]. The mixed findings regarding predictors of practitioner competence and father engagement are not surprising given the diversity in the type of interventions administered and range of practitioners surveyed.

Importantly, this research has not examined predictors of practitioner competence and father engagement in parenting interventions in particular, which is the focus of the present study. In one notable exception, Glynn and Dale [22] conducted a survey with practitioners who delivered parenting interventions. They recruited social workers in New Zealand (*N* = 50) with knowledge of or experience with engaging fathers in parenting interventions, in order to explore practitioners’ views about the most important factors for father engagement. The three most important factors to father engagement identified by practitioners were: qualities of the practitioner, the intervention content and the organizational philosophy. Participants also reported that organizational philosophy and qualities and values of the practitioner were the two factors that were most amenable to change. However, this survey only recruited practitioners with expertise in working with fathers and did not specifically examine predictors of practitioner competence. Therefore, the factors related to practitioner competence within a broader sample of practitioners who deliver parenting interventions has not yet been examined.

In summary, there is limited research on practitioners’ competence and organizational support for working with fathers, especially in relation to delivery of parenting interventions. Given the apparent low rates of father engagement in interventions, practitioners are likely to be an important target for change in enhancing father engagement. However, in order to develop evidence-based training programs and guidelines for practitioners, further research is urgently needed about current practitioner competencies, rates of father engagement, and barriers to father engagement.

The aim of the current study was to survey practitioners who deliver parenting interventions to families in order to examine: (1) characteristics of practitioners who deliver parenting interventions; (2) current rates of father engagement in parenting interventions; (3) practitioners’ attitudes, skills and confidence (competencies) in engaging fathers, and training received in father engagement; (4) perceived barriers to father engagement; and (5) levels of organizational support for father-inclusive practice and strategies organizations use to engage fathers. The current study also examined the practitioner and organizational factors that predicted practitioner competencies and rates of father engagement. On the basis of previous research, there were two hypotheses: Hypothesis 1 was that high levels of practitioner competence would be predicted by greater years of experience as a practitioner and having received training in father engagement, but not male gender or high levels of organizational support; and Hypothesis 2 was that high rates of father engagement would be predicted by greater years of experience as a practitioner, training in father engagement, high levels of practitioner competence and high levels of organizational support, but not male gender.

## Method

### Participants and Recruitment

The survey was completed by 210 participants who each self-identified as a practitioner working with families in Australia to deliver parenting interventions or treatment for child externalizing behaviors. Recruitment involved outreach to a range of government and non-government child and family services around Australia, primarily via email notifications, but also flyers provided at conferences, seminars and meetings. In addition, the survey was promoted via professional bodies such as the Australian Psychological Society and the Australian Fatherhood Research Network. The advertising materials directed interested participants to the project website and the survey was completed online. The survey was available for completion during a 9 month period from August 2015 to April 2016. This survey was part of a national project called *Like Father Like Son*, which seeks to enhance the engagement of fathers in parenting interventions in Australia.

### Measures

The questions included in the survey were selected through a comprehensive review of existing literature on topics such as barriers to participation and practitioner competencies in father engagement. In addition, eight clinical psychologists with experience in delivering parenting interventions with families helped to generate the survey questions, which were then pilot tested with a small convenience sample of practitioners. Based on feedback from the pilot test, items were revised to improve clarity in wording before being included in the final survey.

#### Practitioner Characteristics

Information was collected on practitioners’ gender, profession, years of experience working with families, type of organization they currently worked for, and years of employment with the organization.

#### Current Rates of Father Attendance

Practitioners were asked about rates of father attendance in their organization in the previous calendar year (often, sometimes or rarely) for families with a father present in the home (i.e., able to attend sessions). Practitioners were also asked to estimate the percentage of families referred by fathers in the previous calendar year (with response options of less than 5%, 5–10%, 11–20% and then increasing 10% increments).

#### Practitioner Attitudes and Competencies

Practitioners were asked to rate their overall confidence in engaging fathers on a five-point scale ranging from *extremely confident* (5) to *not at all confident* (1). On the same scale, practitioners were asked to rate their confidence across 13 areas including process issues (e.g., dealing with resistance from fathers, engaging fathers who are reluctant to attend, managing conflict between mothers and fathers), client vulnerabilities (e.g., working with fathers with mental health issues, substance use issues) and knowledge of the literature about father-child relationships. On the same scale, practitioners were asked to rate the frequency of their use of seven specific recommended strategies to engage fathers and keep them engaged (personally inviting fathers to attend, explaining to mothers the importance of engaging fathers, explaining to fathers the importance of being involved, directing equal time and attention to fathers and mothers during sessions, eliciting treatment goals from fathers as well as mothers, problem-solving barriers that prevent fathers from attending, offering separate sessions if fathers cannot attend). Ratings of overall confidence and use of strategies were combined to create a composite measure of practitioner competence in engaging fathers [23]. Practitioners were rated as high in competence if they reported: (1) that they were extremely or very confident about working with fathers; and (2) that they (either always or often) used six practitioner strategies for father engagement (excluding offering separate sessions or phone calls for fathers, as this strategy may be influenced by the service rather than practitioner). Practitioners were rated as lower in competence if they did not meet these criteria.

Two questions were asked about practitioner attitudes: ‘How important do you think it is for fathers to participate in treatment for child issues?’ with responses rated on a five-point scale ranging from *extremely important* (5) through to *not at all important* (1), and ‘Do you believe services or programs are more effective if dads are involved?’, with response options of *yes, no* or *unsure*. Given that practitioner attitudes towards father engagement were on average extremely positive, these items were not included in the composite measure of competence.

#### Practitioner Training

Practitioners were also asked if they had ever received specific training in working with or engaging fathers (either as part of university training or a specific continuing education program). Those who had received training rated its usefulness on a scale ranging from *extremely helpful* (5) to *not at all helpful* (1). Practitioners were also asked whether they would be more likely to participate in web-based or face-to-face training in future, had no preference, or were not likely to participate in either version.

#### Perceived Barriers to Engagement

Practitioners were asked what families or fathers indicate are the barriers to engaging in parenting programs. A total of 20 potential barriers were provided including practical barriers (e.g., problems with transportation, work commitments), personal barriers (e.g., fathers don’t know what the program/service is about, worry about being judged, fathers feel it’s a mother’s role to parent the children), family barriers (e.g., mothers attend the service alone and don’t encourage fathers to participate), and organizational factors (e.g., service doesn’t invite or encourage father participation), with *yes* or *no* response options.

#### Organizational Policies or Practices

Practitioners were asked to what extent they felt their organization supported father-inclusive practice, from *extremely supportive* (5) to *not at all supportive* (1). This variable was collapsed into a binary category due to the limited number of cases in each level/group. Practitioner ratings of *extremely* and *very* were combined into a ‘high’ organizational support category, while ratings of *somewhat* and *not very* were combined into a ‘low’ category (there were no ratings of *not at all*). They were also asked about strategies used by their organization to engage fathers (emphasizing the importance of father attendance at intake, offering sessions outside work hours to enable father attendance, advertising that the program is for fathers as well as mothers, and obtaining data about parenting and child behavior from fathers as well as mothers). Participants responded to these questions on a five point scale from *always* (5) to *never* (1). Practitioners were also asked whether their organization ran father-only groups (with *yes* or *no* response options).

### Procedure

The Human Research Ethics Committee at the University of Sydney provided ethics approval for the study. All flyers and advertisements directed practitioners to a dedicated project website that included the information sheet and consent form for the survey, which was administered using Qualtrics ^TM^ online survey software. Participants gave informed consent before commencing the survey. The questionnaire, which took approximately 15 min to complete, was anonymous and no identifying information was collected.

### Statistical Analysis

Variables were analysed using frequencies and descriptives. Cases with missing data were included in analyses if demographic and professional data were complete and participants had answered any further questions about their work with fathers. To examine whether there were significant differences between those who had only completed demographic data (‘dropouts’) and those who had completed additional survey questions (‘respondents’), a series of independent samples t-tests and Chi square tests was conducted across all available variables.

Two logistic regression analyses were conducted to test predictors of practitioner competence and father engagement. To predict practitioner competence, four independent variables were simultaneously entered into the model: years of experience working with families, practitioner gender, organizational support for father-inclusive practice, and participation in training on engaging fathers. To predict father attendance at programs or services, a multinomial logistic regression analysis was conducted by simultaneously entering the four independent variables from the previous model, plus the dichotomous measure of practitioner competence.

## Results

### Practitioner Characteristics

Of 246 practitioners who began the survey, 210 (85.4%) either completed the survey or answered some questions about their work with fathers. There were no significant differences in demographic or professional characteristics of survey respondents compared to dropouts (*n* = 36). The demographic and professional characteristics of respondents are reported in Table 1. Psychologists (39.0%) and social workers (17.6%) were the most common respondents. Participants had on average 12.6 years (*SD* = 9.8) of experience working with families. Most participants either worked for a non-government organization (40.5%) or government child and family mental health service (24.3%); they had an average of 7 years (*SD* = 6.8) experience working for their current organization, and 75.2% were female.

**Table 1:** Demographic and professional characteristics of participants

Characteristic	Subgroup	Frequency	%
Gender	Female	158	75.2
	Male	52	24.8
Profession	Psychologist	82	39.0
	Social worker	37	17.6
	Family support worker	25	11.9
	Case worker	18	8.6
	Nurse	12	5.7
	Counsellor	8	3.8
	Occupational therapist	5	2.4
	Psychiatrist	3	1.4
	Paediatrician	3	1.4
	Other profession	17	8.1
Organization	Non-government organization	85	40.5
	Government child and family mental health service	51	24.3
	Private practice	33	15.7
	Other government service/organization	25	11.9
	University-based clinic	11	5.2
	Other organization	5	2.4

*N* = 210

### Current Rates of Father Attendance

Most practitioners (87.7%) indicated that less than 30% of families they worked with were referred by the father, and over half said this figure was less than 5%. Regarding families with a father present in the home, 17.2% reported that fathers *often* attended, 53.4% reported fathers *sometimes* attended, and 29.4% of practitioners said that fathers *rarely* attended programs/services.

### Practitioner Competencies

#### Attitudes

The majority of practitioners thought father participation was extremely (79.0%) or very (19.5%) important in treatment for child issues. Only 1.4% endorsed that father participation was somewhat important. The majority of practitioners (92.6%) thought services/programs were more effective if fathers were involved; 6.4% were unsure and 1.0% said they did not believe services/programs were more effective with father involvement.

#### Confidence

Over two-thirds of practitioners reported being very confident (52.7%) or extremely confident (14.1%) in working with fathers; 29.8% were somewhat confident; and 3.4% were not very confident. Practitioner ratings of confidence in working with fathers across a range of specific areas are summarized in Table 2. The three areas in which practitioners were most confident (% extremely or very confident) were: communicating with fathers (80.9%), ability to remain neutral and not side with the mother or father (72.2%), and eliciting fathers’ expectations of treatment and their goals (66.3%). Practitioners were least confident (% extremely or very confident) in: working with fathers who have been violent or abusive (22.4%), working with fathers with substance use issues (26.9%), and dealing with resistance from fathers (39.0%).

**Table 2:** Practitioner confidence [*n* (%)] in engaging and working with fathers

	Extremely	Very	Somewhat	Not very	Not at all
Communicating with fathers	47 (22.9%)	119 (58.0%)	36 (17.6%)	3 (1.5%)	0 (0.0%)
Ability to remain neutral (not side with the mother or father)	42 (20.5%)	106 (51.7%)	50 (24.4%)	7 (3.4%)	0 (0.0%)
Eliciting fathers’ expectations of treatment and their goals	25 (12.2%)	111 (54.1%)	57 (27.8%)	11 (5.4%)	1 (0.5%)
Managing distress from fathers	34 (16.6%)	100 (48.8%)	55 (26.8%)	16 (7.8%)	0 (0.0%)
Working with separated/divorced parents	34 (16.6%)	99 (48.3%)	56 (27.3%)	14 (6.8%)	2 (1.0%)
Understanding fathers’ needs	21 (10.2%)	96 (46.8%)	80 (39.0%)	8 (3.9%)	0 (0.0%)
Knowledge of the literature about father-child relationships	15 (7.3%)	80 (39.0%)	80 (39.0%)	26 (12.7%)	4 (2.0%)
Working with fathers with mental health issues	10 (4.9%)	84 (41.0%)	87 (42.4%)	23 (11.2%)	1 (0.5%)
Engaging fathers who are reluctant to attend	20 (9.8%)	72 (35.1%)	88 (42.9%)	22 (10.7%)	3 (1.5%)
Managing conflict between mothers and fathers	14 (6.8%)	74 (36.1%)	91 (44.4%)	26 (12.7%)	0 (0.0%)
Dealing with resistance from fathers	21 (10.2%)	59 (28.8%)	98 (47.8%)	26 (12.7%)	1 (0.5%)
Working with fathers with substance use issues	4 (2.0%)	51 (24.9%)	88 (42.9%)	54 (26.3%)	8 (3.9%)
Working with fathers who have been violent or abusive	6 (2.9%)	40 (19.5%)	82 (40.0%)	57 (27.8%)	20 (9.8%)

*N* = 205. Rank order based on combined ratings of ‘Extremely’ and ‘Very’

#### Engagement Strategies

Practitioner ratings of strategies used to engage fathers are included in Table 3. In order of frequency (% always or often), these were: explaining to mothers the importance of engaging fathers (84.9%), directing equal time and attention to fathers and mothers (81.0%), explaining to fathers the importance of being involved (77.1%), eliciting treatment goals from fathers as well as mothers (71.3%), personally inviting fathers to attend (70.0%), problem-solving barriers to attendance (52.6%), and offering separate sessions for fathers (49.2%).

**Table 3:** Frequency of use of father engagement strategies [*n* (%)]

	Always	Often	Sometimes	Rarely	Never
Practitioner strategies					
Explaining to mothers the importance of engaging fathers	92 (44.9%)	82 (40.0%)	21 (10.2%)	10 (4.9%)	0 (0.0%)
Directing equal time and attention to fathers and mothers	83 (40.5%)	83 (40.5%)	27 (13.2%)	8 (3.9%)	4 (2.0%)
Explaining to fathers the importance of being involved	75 (36.6%)	83 (40.5%)	38 (18.5%)	9 (4.4%)	0 (0.0%)
Eliciting treatment goals from fathers as well as mothers	69 (33.7%)	77 (37.6%)	38 (18.5%)	17 (8.3%)	4 (2.0%)
Personally inviting fathers to attend (in person or by phone)	50 (24.4%)	75 (36.6%)	56 (27.3%)	21 (10.2%)	3 (1.5%)
Problem-solving barriers that prevent fathers from attending	38 (18.5%)	70 (34.1%)	73 (35.6%)	19 (9.3%)	5 (2.4%)
Where fathers cannot attend, offering separate sessions/phone calls	46 (22.4%)	55 (26.8%)	64 (31.2%)	31 (15.1%)	9 (4.4%)
Service/program strategies					
Obtaining information from fathers as well as mothers	62 (30.5%)	75 (36.9%)	50 (24.6%)	8 (3.9%)	8 (3.9%)
Emphasizing the importance of father attendance at intake	58 (28.6%)	73 (36.0%)	55 (27.1%)	11 (5.4%)	6 (3.0%)
Advertising that the program is for fathers as well as mothers	63 (31.0%)	52 (25.6%)	41 (20.2%)	20 (9.9%)	27 (13.3%)
Offering sessions outside work hours to enable fathers to attend	37 (18.2%)	46 (22.7%)	42 (20.7%)	30 (14.8%)	48 (23.6%)

*N* = 205. Rank order based on combined ratings of ‘Always’ and ‘Often’

#### Composite Measure of Practitioner Competence

Practitioner confidence and use of strategies to engage fathers were combined to create a measure of practitioner competence as described earlier. Approximately one quarter (27.1%) of practitioners were high in competence, while around three-quarters (72.9%) were lower in competence.

### Participation in Training in Father Engagement

Just over one quarter (27.1%) of practitioners reported receiving specific training in working with or engaging fathers. Of those, around three-quarters reported that the training was extremely (29.1%) or very (45.5%) helpful, 23.6% reported it was somewhat helpful and 1.8% reported it was not very helpful.

Only 5.4% of participants reported they were unlikely to participate in either face-to-face or web-based training on father engagement in future. Face-to-face training was selected as the preferred training format by 27.1% of participants, 17.7% reported a preference for web-based training, while around half (49.8%) reported equal likelihood of participating in either format.

### Barriers to Father Engagement

The frequency and percentage of 21 barriers to engaging fathers that practitioners endorsed are displayed in Table 4. The most commonly reported barriers were fathers’ work commitments (81.0%); fathers not having time (55.2%); fathers’ discomfort asking for, or receiving, parenting assistance (53.8%); and fathers feeling that it’s a mother’s role to parent the children (46.7%). The least endorsed barrier was cost of service (4.3%). In addition, 21.9% of practitioners endorsed ‘other’ barriers and provided a text response. Of these, the most prevalent barriers were issues around intimate partner and family violence which may preclude involvement of the father (17.4%), and cultural or language barriers such as lack of culturally appropriate or inclusive services (10.9%).

**Table 4:** Barriers to engaging fathers in programs or services endorsed by practitioners

Type of barrier	Barrier description	Frequency	Percent
Practical barriers	Fathers’ work commitments	170	81.0
	Fathers not having time	116	55.2
	Problems with transport	29	13.8
	No child care	27	12.9
Family factors	Fathers think that problems with their child’s behavior require treatment of the child	90	42.9
	Fathers don’t think their child’s behavior is a problem	79	37.6
	Mothers attend the services alone and don’t encourage fathers to participate	67	31.9
Personal factors	Fathers don’t feel comfortable asking for, or receiving, parenting assistance	113	53.8
	Fathers feel that it’s a mother’s role to parent the children	98	46.7
	Fathers don’t think they need help with their parenting	95	45.2
	Fathers worry about being judged	80	38.1
	Fathers don’t think programs/services are suitable for them	70	33.3
	Fathers don’t know what the program/service is about	63	30.0
	Fathers don’t know whether the program/service is effective	58	27.6
	Previous negative experience with mental health professionals	57	27.1
Organizational factors	Services not available at a convenient time	95	45.2
	Services not held at a convenient location	20	9.5
	Service doesn’t invite or encourage fathers to participate	13	6.2
	Long waiting lists	11	5.2
	Cost of service	9	4.3

*N* = 210

### Organizational Policies or Practices

Over half of practitioners rated their organization as extremely (23.2%) or very (37.4%) supportive of father-inclusive practice, while one-third rated it as somewhat supportive (34.0%). Only 5.4% reported their organization was not very supportive. Practitioners’ reports of strategies used by organizations to engage fathers are included in Table 4. The strategies that practitioners reported to be most commonly used by organizations (% always or often) were: obtaining data from fathers as well as mothers (67.4%), emphasizing the importance of father attendance at intake (64.6%), and advertising that the program is for fathers as well as mothers (56.6%). Only 40.9% of practitioners reported that their organization often or always offered sessions outside working hours. Just under one in five (18.6%) practitioners reported that their service ran father-only groups.

### Predictors of Practitioner Competence and Father Attendance

A logistic regression was conducted to assess the impact of four factors (years of experience, gender, training in father engagement, and organizational support for father-inclusive practice) on level of practitioner competence in working with fathers (see Table 5). The overall model was statistically significant, χ^2^ (4, *N* = 203) = 12.92, *p* < .05. However, the only independent variable to make a unique statistically significant contribution to the model was participation in training in father engagement. That is, practitioners who had received training in father engagement were 2.25 times more likely to be in the high versus low competence category (controlling for other factors in the model).

**Table 5:** Logistic regression model predicting practitioner competence in engaging fathers in programs/services

Variable	B	S.E.	Sig.	Odds ratio	95% Confidence interval
Practitioner years of experience	0.02	0.02	0.23	1.02	0.99–1.05
Gender (male)	0.39	0.37	0.28	1.48	0.72–3.04
Organizational support	0.54	0.35	0.12	1.71	0.86–3.40
Training in father engagement	0.81	0.38	<0.05	2.25	1.13–4.47

*N* = 203

A multinomial logistic regression analysis examined the effect of five predictor variables (years of experience, gender, training in father engagement, organizational support for father-inclusive practice, plus practitioner competence) on father attendance (fathers rarely attended, fathers sometimes attended, fathers often attended) (see Table 6). The overall model was statistically significant, χ^2^ (10, *N* = 197) = 44.15, *p* < .001. For practitioner ratings of ‘sometimes attended’ (relative to ‘rarely’), practitioner years of experience was the only significant predictor. That is, for every additional year of experience, practitioners were 1.05 times more likely to report that fathers sometimes attended relative to rarely attended (controlling for other factors in the model). However, for father attendance ratings of ‘often attended’ (relative to ‘rarely’), there were three significant predictors: years of experience, organizational support, and practitioner competence. Controlling for other factors in the model, for each additional year of experience practitioners were 1.06 times more likely to report that fathers often attended, practitioners with high levels of organizational support were 6.28 times more likely than those with low levels of support to report that fathers often attended, and practitioners with high competence levels were 5.68 times more likely than those with low competence levels to report that fathers often attended.

**Table 6:** Logistic regression model predicting father attendance at programs/services

Category	Variable	B	S.E.	Sig.	Odds ratio	95% Confidence interval
Fathers sometimes attend	Practitioner years of experience	0.05	0.02	<0.05	1.05	1.01–1.10
	Gender (male)	0.03	0.41	0.95	1.03	0.46–2.31
	Organizational support	0.15	0.35	0.66	1.16	0.59–2.29
	Training in father engagement	0.64	0.44	0.14	1.90	0.81–4.47
	Practitioner competence	0.87	0.47	0.06	2.39	0.95–5.99
Fathers often attend	Practitioner years of experience	0.06	0.03	<0.05	1.06	1.01–1.12
	Gender (male)	0.28	0.55	0.60	1.33	0.46–3.87
	Organizational support	1.84	0.62	<0.01	6.28	1.86–21.21
	Training in father engagement	1.07	0.55	0.05	2.91	0.99–8.54
	Practitioner competence	1.74	0.56	<0.01	5.68	1.89–17.04

*N* = 197. The reference category is ‘Fathers rarely attend’

## Discussion

This study reports on a survey of practitioners to examine their competencies in engaging fathers, barriers to engagement of fathers in parenting interventions, and organizational support for father-inclusive practice. While there have been several previous studies with practitioners to examine fathers’ engagement in services and programs, only one study to date has specifically been conducted with practitioners who deliver parenting interventions [22]. The present study involved a survey of practitioners from a broad range of professions and organizations who deliver parenting interventions or treatment for child externalizing problems. Overall, only one in six practitioners reported that fathers *often* attended parenting interventions, with just over half indicating that fathers sometimes attended and just under one-third reporting fathers rarely attended, where there was a father available in the home. This finding is consistent with previous studies and confirms that fathers have low rates of engagement in child-focussed services and interventions [25–27].

Examination of practitioners’ reports of barriers provides insights into their perceptions regarding the key reasons for the low rates of father engagement. The most frequently endorsed barriers were fathers’ work commitments and lack of time, which have been identified as barriers in previous studies with practitioners [22] and fathers [21, 24, 29]. This finding points to the need to provide programs at times that are convenient for fathers, such as evenings and weekends. Despite this, only 40% of practitioners in this study indicated that their organizations frequently provided sessions outside working hours. Providing services outside working hours is unlikely to be feasible for all organizations due to staffing challenges [22], so greater overall flexibility in service provision may also be needed. For example, delivering parenting interventions in the workplace may increase access to services among fathers [30], although this may not achieve the goal of involving both parents in the intervention. Workplaces also need to provide greater flexibility by allowing fathers (and mothers) to attend services for their children during working hours, and research suggests that involvement in parenting interventions may improve both work and family life [31], and therefore may benefit employers as well. Another option for overcoming such barriers is to harness new technologies. For example, evidence from a meta-analytic review (*k* = 12) supports the efficacy of internet-delivered parent training [32]. Importantly, a recent survey of 1000 fathers in the community found that they would be most likely to participate in internet-based interventions [24]. Thus, these programs provide another mode of intervention for families who are unable to attend a face-to-face program.

Around half of practitioners also endorsed barriers such as ‘fathers don’t feel comfortable asking for/receiving parenting assistance’ and ‘fathers feel that it’s a mother’s role to parent the children’, which suggests that help-seeking attitudes and perceptions of masculinity may also be key barriers to participation for some fathers, and this has been identified in qualitative studies with fathers as well [29, 33]. These specific barriers were included in a recent survey of fathers, but were endorsed as a barrier by very few fathers; not feeling comfortable asking for assistance and feeling it was a mother’s role to parent the children were only endorsed by 7% and 3% of fathers respectively [24]. While it is difficult to compare a community sample of fathers with practitioners who largely work in clinical contexts, these differences are striking, and may reflect practitioner overestimation of the extent to which these factors are barriers to father engagement. While in the current study practitioners were asked to indicate whether fathers or families *report* these barriers, it may be that their responses reflect their personal beliefs about these barriers. Alternatively, fathers who attend services delivered by these practitioners may in fact experience these barriers to a greater extent than community-based fathers. In any case, further research with both practitioners and fathers is needed to determine the extent to which help-seeking attitudes and gender-role stereotypes act as barriers to father engagement.

Cost of service was the barrier least endorsed by practitioners. In contrast, a recent survey of fathers found that one in five rated cost of service as a barrier to participation, and it was the most frequently endorsed barrier (along with work commitments) from more than twenty barriers listed [24]. It is possible that practitioners may not be aware of cost as a barrier to participation, perhaps because families who attend are in general able to afford treatment. Alternatively, it is possible that the practitioners in this study largely provided interventions free of charge or at low cost which would explain the differences in perceptions between these practitioners and fathers. Although this study did not ask practitioners if their program incurred a cost, given that almost two-thirds of practitioners worked for a non-government organization or a government family mental health service, it could be assumed that most would not charge a fee for service.

Just under one in three practitioners endorsed the barrier that mothers attended services alone and did not encourage fathers to participate. This suggests that for some families, mothers may play a key role in either facilitating or preventing fathers from engaging in treatments or services. The term ‘maternal gatekeeping’, which was originally used to describe whether mothers encourage or discourage father involvement in domestic or child care responsibilities [34–36], has also been used to describe the extent to which mothers facilitate the participation of fathers in parenting interventions [22]. There has been no empirical research on this issue to date, so the extent to which mothers’ attitudes and behaviors influence father participation is unknown. However, for families where only the mother attends an initial appointment, practitioners could benefit from eliciting mothers’ attitudes towards father engagement, emphasizing the importance of father engagement to the success of the intervention and maintenance of treatment gains, and exploring mothers’ willingness to facilitate father engagement. In other words, practitioners need to develop confidence and skills in engaging fathers indirectly through mothers, as well as engaging them directly.

Practitioner attitudes towards father engagement were overwhelmingly positive, with over 9 in 10 practitioners reporting a belief that interventions are more effective if fathers are involved. While on the one hand this finding suggests that practitioners are already aware of the importance of fathers to the success of parenting interventions, it is also possible that practitioners who held positive views of fathers were more likely to participate in this study, indicating potential sampling bias. It was also encouraging to find that most practitioners felt either very or extremely confident in engaging fathers. However, when examining competence, which combined both confidence and frequent use of strategies to engage fathers, only one in four were classified as high in competence, which indicates that practitioners’ skills in engaging fathers may need to be enhanced.

In terms of confidence with specific skills or client vulnerabilities, the areas where practitioners were least confident were working with fathers who have been violent or abusive, and working with fathers who have substance use issues, with only around a quarter of practitioners indicating they were very or extremely confident in dealing with these issues. It is not surprising that these are the areas in which practitioners felt least confident, as they are challenging clinically, and are possibly areas in which practitioners receive little or no training. However, it is important to bear in mind that these findings may not specifically relate to fathers, and practitioners may also be low in confidence when working with mothers who have been violent or those using substances. In addition, less than half of practitioners indicated they were very or extremely confident in topics such as dealing with resistance from fathers, managing conflict between mothers and fathers, and engaging fathers who are reluctant to attend. Together these findings suggest that there may be a need for more training in therapeutic strategies to address these issues. Only around one quarter of practitioners indicated that they had participated in a training program focused on father engagement, which suggests that they may not be widely available. Indeed, other researchers have described the limited provision of training and professional development in father engagement for professionals working with families [1, 28], and such training is not usually part of undergraduate or postgraduate courses for health professionals [37].

In terms of strategies that practitioners used to engage fathers, ‘explaining to mothers the importance of engaging fathers’ was the most frequently used strategy overall (and used more frequently than ‘personally inviting fathers to attend’), providing further evidence that mothers may often be the initial contact point and the ‘gatekeepers’ for engagement. This is also supported by the very low rates of father referrals to the service or program, although we did not ask about rates of mother referral to enable a comparison. This finding suggests that fathers are not the primary instigators of help-seeking. While there is a lack of research on gender differences in help-seeking for child mental health problems, previous research suggests that the presence of a father in the home may inhibit children receiving mental health treatment, possibly because fathers may be more resistant to treatment [38]. There were a number of relatively easy-to-implement strategies that less than two-thirds of practitioners reported using frequently, such as directly inviting fathers to attend, and problem-solving barriers to father attendance. As highlighted by Duhig et al. [25], it is critical for practitioners to talk to fathers directly where possible, in order to invite their participation, educate them about the program, and emphasize the importance of their involvement (and that of the core parenting team) to the success of the intervention [39]. Thus, future training programs should include active skills training for practitioners in inviting fathers to participate and problem-solving barriers to attendance, as these are likely to be important, easy-to-implement strategies for enhancing rates of father engagement.

Two-thirds of practitioners reported that their organization frequently collects data from fathers as well as mothers. This finding is relatively positive, given that a lack of outcome measures for fathers has been consistently highlighted as a key gap in research on parenting interventions [6, 8]. Obtaining assessment information (such as standardized questionnaires about child behavior) from mothers, fathers and other relevant informants (such as teachers) is critical to treatment conceptualization, because of the unique information provided by each informant [25]. In addition, repeated assessment at post-intervention is important for determining intervention effectiveness, especially as there is evidence that fathers may benefit less from parenting interventions (in terms of level of change in parenting and child behavior) than mothers [4, 6, 10]. Practitioner training should emphasize the need for ongoing assessment of all available caregivers, in an effort to enhance treatment conceptualization and evaluation of treatment effectiveness. Less than two-thirds of practitioners indicated that their organization frequently used strategies such as advertising that the program is for fathers as well as mothers, and highlighting the importance of fathers at intake. These strategies are relatively easy to implement and should be included in future training programs in an effort to increase father-inclusive practice in organizations.

Just under one in five practitioners reported that their organization runs father-only groups. There may be some circumstances where father-only groups are appropriate, such as for single-father families or gay fathers. However, practitioners should be cautious in providing father-only groups in two-parent families (as they should in relation to mother-only groups), due to the possibility of reduced effectiveness, or even unintended negative effects. For example, a randomized controlled trial (RCT) of a father-only parenting intervention found the intervention resulted in significant decreases in child behavior problems as rated by fathers relative to a control group, but mothers’ ratings increased, and father ratings of the quality of the partner relationship appeared to decrease in the intervention group [40]. Similarly, an RCT of a preventive parenting intervention that randomized parents to a father-only group, a couple group or a control condition, found that the father-only group showed declines in relationship satisfaction over time as rated by both parents [35]. These findings suggest that exclusion of mothers may have deleterious effects on the partner relationship and even on ratings of child behavior. While these findings highlight the importance of not actively excluding a parent from an intervention, it is also important to note that both parents may not necessarily have to participate directly in the intervention to experience benefits. It is possible that indirect participation can be effective, for example when one parent participates and communicates the information to the second caregiver [20], yet there is scant research about indirect participation and the circumstances under which it is effective.

This study also examined practitioner and organizational factors that predicted practitioner competencies and rates of father engagement. There was partial support for Hypothesis 1, in that participation in training predicted higher practitioner competence, and both male gender and high levels of organizational support were not significant predictors. While these findings are broadly consistent with previous research [26, 28], contrary to expectation and previous research [28], experience did not predict practitioner competence. This suggests that competence is not necessarily dependent on greater experience, and participation in training may be important. This has recently been shown in a study reporting that a one-day father-focussed workshop for health visitors in the UK resulted in improved practitioner knowledge, attitudes and behaviors related to engagement of fathers [41].

More support was found for Hypothesis 2 in that moderate father attendance (sometimes vs. rarely) was significantly predicted by greater years of experience, and high father attendance (often vs. rarely) was predicted by greater years of experience, high competence, and higher levels of organizational support for father-inclusive practice, findings which are also consistent with previous research [25, 26, 28]. Male gender did not emerge as a significant predictor of father engagement, which was in keeping with the findings of Duhig et al. [25] and Lazar et al. [26], in relation to father engagement in two-parent families. Contrary to expectations, the current study did not find an association between participation in training on the topic of father engagement and higher rates of father attendance, although this approached significance. Previous research has found training and/or professional development activities such as completion of seminars, courses and reading to be associated with increased father attendance [25, 26], yet these studies did not ask about participation in training specifically on the topic on father engagement. Thus, further research is needed, especially as one study found that a brief training program on strategies to enhance parental engagement resulted in significant improvements in parental attendance and reductions in attrition in a community mental health clinic [42], although this study did not specifically focus on fathers. Further research examining the association between training and attendance should aim to use experimental designs to evaluate whether training in skills to enhance father engagement results in significant improvements in rates of father engagement over time, and what potential mechanisms may play a role in this relationship.

In relation to the finding that a high level of father attendance was associated with greater organizational support, other research has found that practitioners perceive organizational support to be an important factor for father engagement, and one of the factors that is most feasible to change [22]. This suggests that organizations should increase their support for father-inclusive practice, and further research should explore methods for achieving this. This may include implementing organizational policies, training programs, staff supervision and/or professional development activities focussed on enhancing father-inclusive practice. Training or education programs should aim to include those in managerial positions, in order to bring about systems-level change. While most practitioners reported that their organization was very or extremely supportive of father-inclusive practice, one-third indicated that their organization was only somewhat supportive, again indicating the need for increased organizational support.

There are a number of limitations that should be considered when interpreting these findings. First, as previously mentioned, our sample of practitioners may not be representative of practitioners who deliver parenting interventions in the community. We did not obtain data from practitioners about geographic location and therefore we cannot say whether our sample was nationally representative. In addition, our sample had generally positive attitudes towards father engagement and had, on average, over a decade of experience in working with families. Furthermore, one quarter of participants in the survey were male, and this proportion may be higher than the predominantly female workforce who work with families, although it is consistent with the proportion of psychologists in Australia who are male [43]. As the survey was voluntary, it is possible that respondents were motivated to participate as they had more experience with fathers and/or an existing interest in engaging fathers. Future research should aim to include a more representative sample of practitioners, including practitioners with fewer years of experience working with families. Second, it is important to keep in mind that the regression analyses indicated only associations between variables, rather than causal relationships, and these associations may not be in the expected direction. For example, rather than training resulting in high levels of competence, it is possible that those higher in competence may be more likely to participate in training. Future experimental studies are needed to clarify the causal relationships between variables examined in this study. Third, there were a number of questions that were not included in the current research which may have shed more light on the findings, such as cost of the service and rates of mother referral. Fourth, as practitioners were not asked about their skills and confidence in engaging mothers, it is not possible to know whether competencies and perceptions of barriers to engagement differ for engaging mothers versus fathers. Finally, while we contrasted the findings of this practitioner survey with a recent survey of community fathers, to better compare fathers’ and practitioners’ perceptions, it would be worthwhile to include a sample of fathers and practitioners from the same service or program in future research studies.

While further research is needed to replicate these results with more representative samples of practitioners who work with families, this survey is an important step towards understanding current levels of practitioner competencies, rates of father engagement and barriers to father engagement. As such, there are several implications of the findings of this study to the development of policies and practices to both increase practitioner competencies and promote the engagement of fathers in evidence-based parenting interventions. First, there is a need to provide and evaluate training programs on the topic of engaging fathers, since a minority of practitioners reported participating in such training, and participation was associated with higher levels of competence. It is important that any future training programs are evaluated using experimental trials, where possible, and examine the impact on rates of father participation over time. Second, practitioners could increase their use of fairly simple engagement strategies such as directly inviting fathers to attend and problem-solving barriers to attendance. Third, at the organizational level, there is opportunity to increase strategies such as emphasizing the importance of father attendance at intake and advertising that the program is for both fathers and mothers. There is also a clear need for flexible service delivery and for programs to be available outside of working hours, and for assessment data to be collected from both parents throughout intervention. Organizational support appears to be important for achieving high rates of father engagement, and training provided should include strategies for bringing about changes at the organizational level.

The findings from this study have been used to inform the development of a National Training Program in Father Engagement in Australia, as part of the *Like Father Like Son* project. This project aims to enhance the engagement of fathers in evidence-based parenting interventions at a national level. This training, which will be available both online and face-to-face, will be provided at no cost to participants, and will include topics highlighted in this study, including how to engage reluctant fathers and how to build confidence in managing conflict in sessions.

## Summary

This study reported on the results of an online survey of practitioners who work with families in Australia to deliver parenting interventions or treatment for child externalizing problems. The results indicated that practitioners have a high level of self-reported confidence in engaging fathers, but only one in three had participated in training on father engagement and levels of father attendance at services were low. Practitioner attitudes towards father engagement were overwhelmingly positive. However, only one quarter of practitioners were classified as high in competence, a measure which combined both confidence and frequent use of strategies to engage fathers. These findings suggest that there is room to enhance practitioners’ skills in engaging fathers and a need to provide and evaluate training programs on the topic of engaging fathers, especially as participation in training was associated with higher levels of practitioner competence. Organizational support for father-inclusive practice also appears to be important for achieving high rates of father engagement, and further research should explore strategies for bringing about organizational change.

## References

[1] Gordon DM, Oliveros A, Hawes SW, Iwamoto DK, Rayford BS (2012). Engaging fathers in child protection services: a review of factors and strategies across ecological systems. Child Youth Serv Rev.

[2] Maxwell N, Scourfield J, Featherstone B, Holland S, Tolman R (2012). Engaging fathers in child welfare services: a narrative review of recent research evidence. Child Fam Soc Work.

[3] Zanoni L, Warburton W, Bussey K, McMaugh A (2013). Fathers as ‘core business’ in child welfare practice and research: an interdisciplinary review. Child Youth Serv Rev.

[4] Fletcher R, Freeman E, Matthey S (2011). The impact of behavioural parent training on fathers’ parenting: a meta-analysis of the Triple P-Positive Parenting Program. Fathering.

[5] Flippin M, Crais ER (2011). The need for more effective father involvement in early autism intervention a systematic review and recommendations. J Early Interv.

[6] Panter-Brick C, Burgess A, Eggerman M, McAllister F, Pruett K, Leckman JF (2014). Practitioner review: engaging fathers-Recommendations for a game change in parenting interventions based on a systematic review of the global evidence. J Child Psychol Psychiatry.

[7] Smith TK, Duggan A, Bair-Merritt MH, Cox G (2012). Systematic review of fathers’ involvement in programmes for the primary prevention of child maltreatment. Child Abuse Rev.

[8] Tiano JD, McNeil CB (2005). The inclusion of fathers in behavioral parent training: a critical evaluation. Child Fam Behav Ther.

[9] Epstein RA, Fonnesbeck C, Potter S, Rizzone KH, McPheeters M (2015). Psychosocial interventions for child disruptive behaviors: a meta-analysis. Pediatrics.

[10] Sanders MR, Kirby JN, Tellegen CL, Day JJ (2014). The triple P-positive parenting program: a systematic review and meta-analysis of a multi-level system of parenting support. Clin Psychol Rev.

[11] Lundahl BW, Tollefson D, Risser H, Lovejoy MC (2008). A meta-analysis of father involvement in parent training. Res Soc Work Pract.

[12] Fabiano GA (2007). Father participation in behavioral parent training for ADHD: review and recommendations for increasing inclusion and engagement. J Fam Psychol.

[13] Budd KS, O’Brien TP (1982). Father involvement in behavioral parent training: an area in need of research. Behav Ther.

[14] Bagner DM, Eyberg SM (2003). Father involvement in parent training: when does it matter?. J Clin Child Adolesc Psychol.

[15] Webster-Stratton C (1985). The effects of father involvement in parent training for conduct problem children. J Child Psychol Psychiatry.

[16] Rhoades KA (2008). Children’s responses to interparental conflict: a meta-analysis of their associations with child adjustment. Child Dev.

[17] Cosson B, Graham E (2012). ‘I felt like a third wheel’: fathers’ stories of exclusion from the parenting team. J Fam Stud.

[18] Deinhart A (1998). Reshaping fatherhood: the social construction of shared parenting.

[19] Lamb ME (2004). The role of the father in child development.

[20] Piotrowska PJ, Tully LA, Lenroot R, Kimonis ER, Hawes DJ, Moul C, Frick PJ, Anderson V, Dadds MR (2016) Mothers, fathers, and parental systems—a conceptual model of parental engagement in programs for child mental health: connect, attend, participate, enact (CAPE). Clin Child Fam Psychol Rev. doi:10.1007/s10567-016-0219-9 10.1007/s10567-016-0219-9 PMC5487721 27914017

[21] Frank TJ, Keown LJ, Dittman CK, Sanders MR (2015). Using father preference data to increase father engagement in evidence-based parenting programs. J Child Fam Stud.

[22] Glynn L, Dale M (2015). Engaging dads: enhancing support for fathers through parenting programmes. Aotearoa New Zealand Soc Work.

[23] Rodolfa E, Bent R, Eisman E, Nelson P, Rehm L, Ritchie P (2005). A cube model for competency development: implications for psychology educators and regulators. Prof Psychol.

[24] Tully LA, Piotrowska PJ, Collins DJ, Mairet K, Black N, Kimonis ER et al. (in press) Optimizing child outcomes from parenting interventions: fathers’ experiences, preferences and barriers to participation. BMC Public Health 10.1186/s12889-017-4426-1 PMC5463495 28592244

[25] Duhig AM, Phares V, Birkeland RW (2002). Involvement of fathers in therapy: a survey of clinicians. Prof Psychol.

[26] Lazar A, Sagi A, Fraser MW (1991). Involving fathers in social services. Child Youth Serv Rev.

[27] Scourfield J, Cheung SY, Macdonald G (2014). Working with fathers to improve children’s well-being: results of a survey exploring service provision and intervention approach in the UK. Child Youth Serv Rev.

[28] Fletcher R, Freeman E, Ross N, St George J (2013). A quantitative analysis of practitioners’ knowledge of fathers and fathers’ engagement in family relationship services. Aust Disp Resol J.

[29] Walters J, Tasker F, Bichard S (2001). ‘Too busy’? Fathers’ attendance for family appointments. J Fam Ther.

[30] Sanders MR, Haslam DM, Calam R, Southwell C, Stallman HM (2011). Designing effective interventions for working parents: a web-based survey of parents in the UK workforce. J Child Serv.

[31] Sanders MR, Stallman HM, McHale M (2011). Workplace triple P: a controlled evaluation of a parenting intervention for working parents. J Fam Psychol.

[32] Nieuwboer CC, Fukkink RG, Hermanns JM (2013). Online programs as tools to improve parenting: a meta-analytic review. . Child Youth Serv Rev.

[33] Dolan A (2014). ‘I’ve learnt what a dad should do’: the interaction of masculine and fathering identities among men who attended a ‘dads only’parenting programme. Sociology.

[34] Allen SM, Hawkins AJ (1999). Maternal gatekeeping: mothers’ beliefs and behaviors that inhibit greater father involvement in family work. J Marriage Fam.

[35] Cowan PA, Cowan CP, Pruett MK, Pruett K, Wong JJ (2009). Promoting fathers’ engagement with children: preventive interventions for low-income families. J Marriage Fam.

[36] McBride BA, Brown GL, Bost KK, Shin N, Vaughn B, Korth B (2005). Paternal identity, maternal gatekeeping, and father involvement. Fam Relat.

[37] Fletcher R, May C, St George J, Stoker L, Oshan M (2014). Engaging fathers: evidence review.

[38] Zimmerman FJ (2005). Social and economic determinants of disparities in professional help-seeking for child mental health problems: evidence from a national sample. Health Serv Res.

[39] Hecker LL (1991). Where is dad? 21 ways to involve fathers in family therapy. J Fam Psychother.

[40] Helfenbaum-Kun ED, Ortiz C (2007). Parent-training groups for fathers of head start children: a pilot study of their feasibility and impact on child behavior and intra-familial relationships. Child Fam Behav Ther.

[41] Humphries H, Nolan M (2015). Evaluation of a brief intervention to assist health visitors and community practitioners to engage with fathers as part of the healthy child initiative. Prim Health Care Res Dev.

[42] Watt BD, Dadds MR, Best D, Daviess C (2012). Enhancing treatment participation in CAMHS among families of conduct problem children: effectiveness study of a clinician training programme. Child Adolesc Ment Health.

[43] Australian Institute of Health and Welfare (2014) National Workforce Data Set http://www.aihw.gov.au/workforce-data/

